# Prognostic significance of pretreatment systemic immune-inflammation index in patients with prostate cancer: a meta-analysis

**DOI:** 10.1186/s12957-022-02878-7

**Published:** 2023-01-05

**Authors:** Buwen Zhang, Tao Xu

**Affiliations:** 1 Department of Oncology, Changxing People’s Hospital, Huzhou, 313199 Zhejiang China; 2 Department of Urology, Changxing People’s Hospital, Huzhou, 313199 Zhejiang China

**Keywords:** Systemic immune-inflammation index, Prostate cancer, Meta-analysis, Prognostic factors, Clinical use

## Abstract

**Background:**

The SII (systemic immune-inflammation index) has been extensively reported to have a prognostic value in prostate cancer (PCa), despite the unconformable results. The purpose of this meta-analysis is to quantify the effect of pretreatment SII on survival outcomes in patients with PCa.

**Methods:**

The following databases were searched: Web of Science, Cochrane Library, PubMed, Embase, and China National Knowledge Infrastructure (CNKI). For exploration of the SII’s correlations with the overall survival (OS) and the progression-free survival/biochemical recurrence-free survival (PFS/bRFS) in PCa, the pooled hazard ratios (HRs) were assessed within 95% confidence intervals (CIs).

**Results:**

The present meta-analysis covered 10 studies with 8133 patients. Among the PCa population, a high SII was linked significantly to poor OS (*HR* = 2.63, 95% *CI* = 1.87–3.70, *p* < 0.001), and worse PFS/bRFS (*HR* = 2.49, 95% *CI* = 1.30–4.77, *p* = 0.006). However, a high SII was not linked significantly to T stage (*OR* = 1.69, 95% *CI* = 0.86–3.33, *p* = 0.128), the metastasis to lymph node (*OR* = 1.69, 95% *CI* = 0.69–4.16, *p* = 0.251), age (*OR* = 1.41, 95% *CI* = 0.88–2.23, *p* = 0.150), or the Gleason score (*OR* = 1.32, 95% *CI* = 0.88–1.96, *p* = 0.178).

**Conclusions:**

For the PCa sufferers, the SII might be a promising prognostic biomarker, which is applicable to the high-risk subgroup identification, and provide personalized therapeutic strategies.

**Supplementary Information:**

The online version contains supplementary material available at 10.1186/s12957-022-02878-7.

## Background

Apart from being the 2nd commonly diagnosed carcinoma, prostate cancer (PCa) also represents the 5th leading cause of carcinoma-associated mortality among males globally [1]. Based on GLOBOCAN 2020 estimates, there were 1,414,259 new PCa cases and 375,304 PCa-associated deaths in 2020 around the world [1]. The global incidence of PCa varies more than 25-fold, with a higher prevalence in Western countries and a lower prevalence in Asian countries [2]. The last few decades have witnessed an elevation in the global PCa incidence [3]. Its prognosis is heterogeneous, according to tumor stage. Most PCa cases have localized disease, and the 5-year rate of survival is nearly 100% in these populations. However, the patients with metastatic castration-resistant PCa (mCRPC) have poor prognosis, whose median time of survival is 24 months and 5-year rate of survival 30% [4, 5]. Prognostic biomarkers are important for improving the survival outcomes of patients with PCa [6]. For example, a recent study (PRIMERA trial) including 44 patients revealed that androgen receptor (AR), prostate-specific antigen (PSA), and prostate-specific membrane antigen (PSMA) expression in circulating tumor cells (CTC)+ had no significant impact on PSA drop and survival in mCRPC patients [7]. The PRIMERA trial validated the predictive importance of CTC detection in mCRPC patients as a result [7]. Hence, identification of novel biomarkers and treatment targets is imperative, in order to enhance the prognosis for patients with PCa.

As indicated by growing evidence, the systemic inflammatory reactions are significant determinants of cancer development and survival outcomes in various cancer types [8]. Many serum inflammatory parameters, including the ratios of neutrophils/lymphocytes [9], platelets/lymphocytes [10], C-reactive proteins/albumin [11], and the SII (systemic immune-inflammation index) [12], have been reported to be effective prognostic markers in different cancer types. The SII was calculated as follows: platelet quantity × neutrophil quantity/lymphocyte quantity. Its role as a prominent prognostic biomarker has been demonstrated in numerous types of carcinomas, such as the hepatocellular [13], pancreatic [14], breast [15], and non-small cell lung [16] carcinomas. Despite the prior explorations on SII’s prognostic significance among the PCa population, unconformable results have been yielded [17–26]. High SII in PCa has been considered a valid prognostic biomarker for the poor outcome by several researchers [19, 21, 24], whereas others have denied this association [20]. Hence, the objective of the present meta-analysis is to evaluate SII’s prognostic value in PCa based on current evidence.

## Materials and methods

### Study guideline and ethics approval

The present meta-analysis was carried out as per the guidelines of the Preferred Reporting Items for Systematic Reviews and Meta-Analyses (PRISMA) [27]. The PRISMA checklist was shown in supplemental file 1. This meta-analysis has been registered with INPLASY (Registration No. INPLASY2022110155) and is available from https://inplasy.com/inplasy-2022-11-0155/. Being a literature-based study in nature, ethical approval was unnecessary; moreover, no data containing individual patient information were used.

### Data sources and literature search

The following electronic databases were searched thoroughly: Web of Science, Cochrane Library, PubMed, Embase, and China National Knowledge Infrastructure (CNKI). The search duration was from the databases’ inception to November 27, 2022. The search heading terms and keywords included “systemic immune-inflammation index,” “SII,” “prostate cancer,” “PCa,” “prostate carcinoma,” and “prostate neoplasm.” All searches used both subject headings of Medical Subject Headings (MeSH) terms and free test words. The detailed literature strategies for each database are shown in supplemental file 2. No language restrictions were applied. The reference lists were manually searched to find eligibility records.

### Selection criteria

The inclusion criteria were identified according to the PICOS (population, intervention, comparator, outcomes, and study) criteria. The inclusion criteria were formulated as shown below:

(i) P (population): Patients whose PCa was confirmed pathologically
(ii) I (intervention): The SII level was examined pretreatment for PCa patients, and studies identified a cutoff value of SII for stratifying patients as low/high SII.
(iii) C (comparator): PCa patients with low SII level
(iv) O (outcomes): Studies reported association between SII and PCa survival outcomes; presented any of such survival outcomes as bRFS (biochemical recurrence-free survival), RFS (recurrence-free survival), DFS (disease-free survival), PFS (progression-free survival), and OS (overall survival); and provided HRs (hazard ratios) and corresponding 95% CIs (confidence intervals) for survival outcomes or provided sufficient data to calculate them.
(v) S (study design): Cohort studies, including prospective and retrospective cohorts published in English or Chinese.

Studies were excluded when any of the following criteria was satisfied: (i) case reports, meeting abstracts, reviews or letters, (ii) studies with overlapping patients, (iii) studies with inadequate data for making HR and 95% CI estimations, and (iv) nonhuman studies.

### Data extraction and quality assessment

All of the retrieved studies were assessed by 2 independent investigators (B. Z. and T. X.), who were also responsible for extracting information based on a designated form. Disputes were all resolved through negotiation until a consensus was reached. Information extracted included the name of first author, country, age, year of publication, sample size, research design, study duration, metastatic status of disease, therapeutic management, follow-up, SII cutoff, method for cutoff selection, quantity of patients having low/high SII, models of survival analysis, survival endpoints, study center, and HRs with 95% CIs. The methodological quality of enrolled studies was assessed by two reviewers (B. Z. and T. X.) independently on the NOS (Newcastle–Ottawa scale) [28], which achieves quality evaluation from 3 dimensions: selection, comparability, and outcome of interest. The NOS scores varied between 1 and 9 points, and the quality of studies was considered high when the NOS scores ≥ 6.

### Statistical analysis

SII’s prognostic significance for OS and PFS was assessed by estimating the pooled HRs with 95% CIs. For the evaluation of inter-study statistical heterogeneity, the *χ*^2^-based *Q*-test combined with Higgins’ *I*^2^ test was employed. Inter-study heterogeneity was considered significant when the *p*-value of *Q*-test (Ph) < 0.10 and *I*^2^ > 50%; accordingly, we adopted the random-effects model. In other cases, a fixed-effects model was utilized. Further exploration was made via the subgroup analysis. The association of SII with the clinicopathological traits of PCa was examined through computation of ORs (odds ratios) and 95% CIs. Sensitivity analysis was used to examine the stability of the results. Possible publication bias was detected by utilizing the Egger’s test in conjunction with Begg’s funnel plot. All of the statistical analyses were made via the Stata 12.0 (StataCorp, TX, USA), and *p*-values of < 0.05 were regarded as statistically significant.

## Results

### Study selection process

**Fig. 1:**
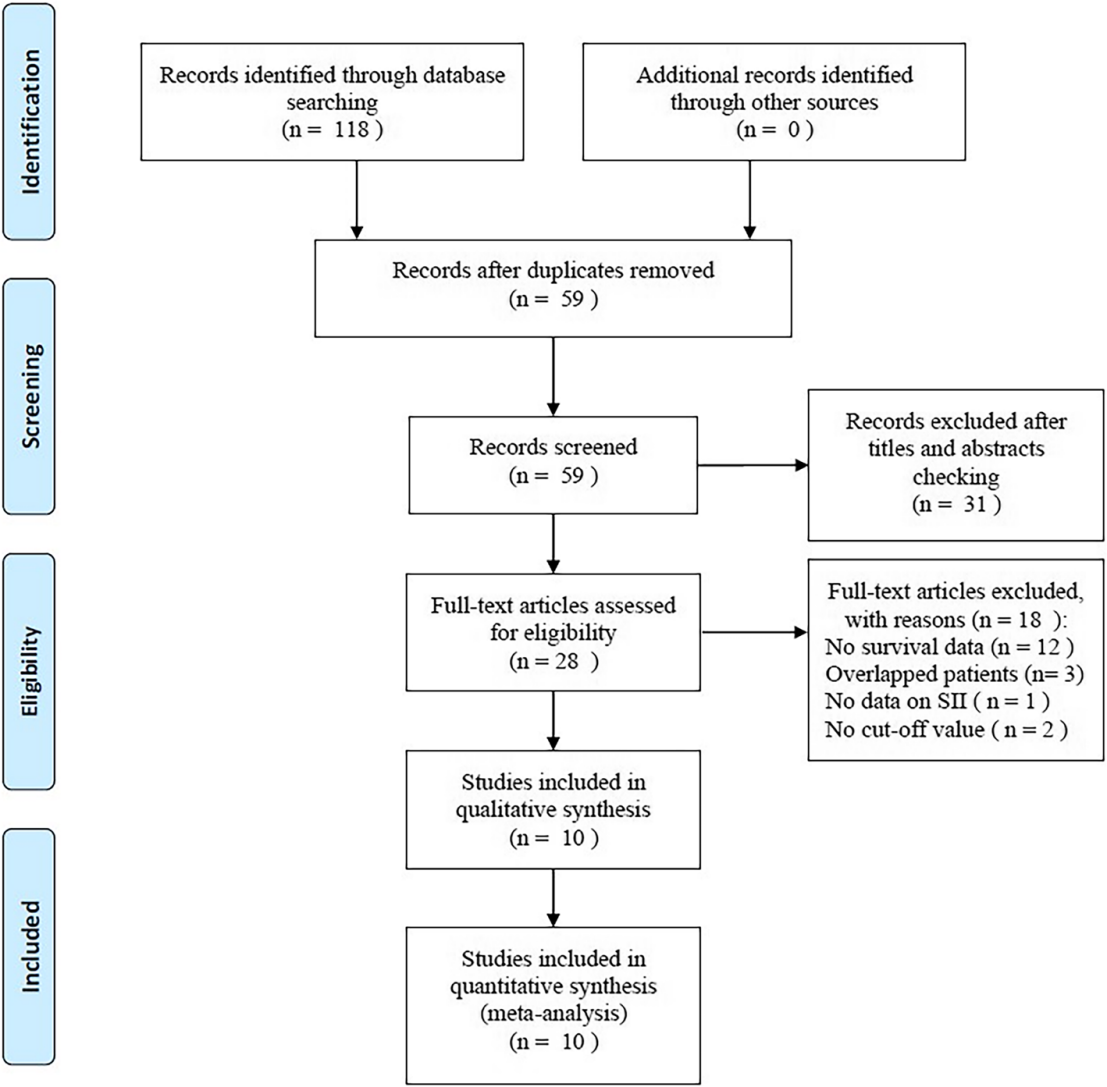
Flowchart for selection of studies

Figure 1 displays a PRISMA flowchart for screening studies. A total of 118 records were identified upon the initial literature retrieval, and after elimination of duplicate records, 59 studies were retained. Next, 31 of these 59 studies were excluded upon examination of their titles and abstracts, and the remaining 28 studies were subjected further to the full-text examination. Thereafter, 18 studies were eliminated due to absence of data on survival (12 studies), recruitment of overlapping patients (3 studies), no cutoff value (2 studies), and the absence of data on SII (1 study). Finally, the number of studies included in the present meta-analysis totaled 10, involving 8133 patients [17–26].

### Characteristics of the included studies

Table 1 details the basic traits of the enrolled studies [17–26], which were published from 2016 to 2022. The size of samples varied from 80 to 6039, with a median value of 204.5. Four studies were conducted in China [19, 22, 23, 25], two in Italy [17, 21], and one each in Spain [18], Japan [20], Poland [26], and Austria [24]. Language of publication was English for 9 studies [17–22, 24–26] whereas was Chinese for 1 study [23]. Six studies included mCRPC patients [17–22], and four studies included patients with nonmetastatic PCa [23–26]. Nine studies were retrospective [17, 19–26] and one was a prospective trial [18]. Eight studies were single-center studies [18–23, 25, 26], while two were multicenter studies [17, 24]. The SII cutoff varied between 200 and 900, with a median of 576. Eight studies offered data concerning the SII–OS correlation [17–23, 26], and two studies presented data on the correlation between the SII and PFS [19, 20], and two studies reported the data on connection between SII and bRFS [24, 25]. Variation scope of NOS scores was 6–9 for the enrolled studies, exhibiting a median of 8, suggesting that the quality of entire enrolled studies was high (Table 1).

**Table 1:** Basic characteristics of included studies

Author	Year	Country	Sample size	Age (years) Median (range)	Study design	Study duration	Metastatic status	Treatment	Follow-up (months)	Cut-off value	Cut-off selection	Patients of low/high SII	Survival endpoints	Survival analysis type	Study center	NOS score
Bauckneht, M.	2021	Italy	519	74 (50–90)	Retrospective	2013–2020	mCRPC	Radium-223	10.7	768.8	ROC curve	260/132	OS	Multivariate	Multicenter	7
Donate-Moreno, M. J.	2020	Spain	80	72.7	Prospective	2014–2018	mCRPC	Hormone therapy + chemotherapy	19	535	Literature	47/33	OS	Univariate	Single center	9
Fan, L.	2018	China	104	72	Retrospective	2013–2017	mCRPC	Hormone therapy + chemotherapy	19.2	200	ROC curve	NR	OS, PFS	Multivariate	Single center	6
Kobayashi, H.	2022	Japan	144	71	Retrospective	2008–2018	mCRPC	Chemotherapy	3–36	636	Median value	72/72	OS, PFS	Multivariate	Single center	7
Lolli, C.	2016	Italy	230	74 (45–90)	Retrospective	2011–2015	mCRPC	Hormone therapy + chemotherapy	18	535	X-tile software	98/132	OS	Univariate	Single center	8
Man, Y. N.	2019	China	179	70 (51–88)	Retrospective	2010–2018	mCRPC	Hormone therapy + chemotherapy	24 (2–118)	535	Literature	85/94	OS	Multivariate	Single center	8
Pan, Z.	2020	China	126	64 (43–76)	Retrospective	2013–2015	Nonmetastatic PCa	Radical prostatectomy	To June 2019	617	Mean value	72/54	OS	Multivariate	Single center	7
Rajwa, P.	2021	Austria	6,039	61	Retrospective	2000–2011	Nonmetastatic PCa	Radical prostatectomy	44	620	ROC curve	4,341/1698	bRFS	Multivariate	Multicenter	8
Wang, S.	2022	China	291	66.13	Retrospective	2014–2019	Nonmetastatic PCa	Radical prostatectomy	48	528.54	ROC curve	129/162	bRFS	Multivariate	Single center	8
Zapala, P.	2022	Poland	421	65	Retrospective	2012–2018	Nonmetastatic PCa	Radical prostatectomy	69	900	ROC curve	203/218	OS	Multivariate	Single center	8

*OS* overall survival, *PFS* progression-free survival, *SII* systemic immune-inflammation index, *mCRPC* metastatic castration-resistant prostate cancer, *ROC* receiver operating characteristic, *NOS* Newcastle-Ottawa scale, *NR* not reported, *PCa* prostate cancer

### Prognostic of SII for OS in PCa

**Fig. 2:**
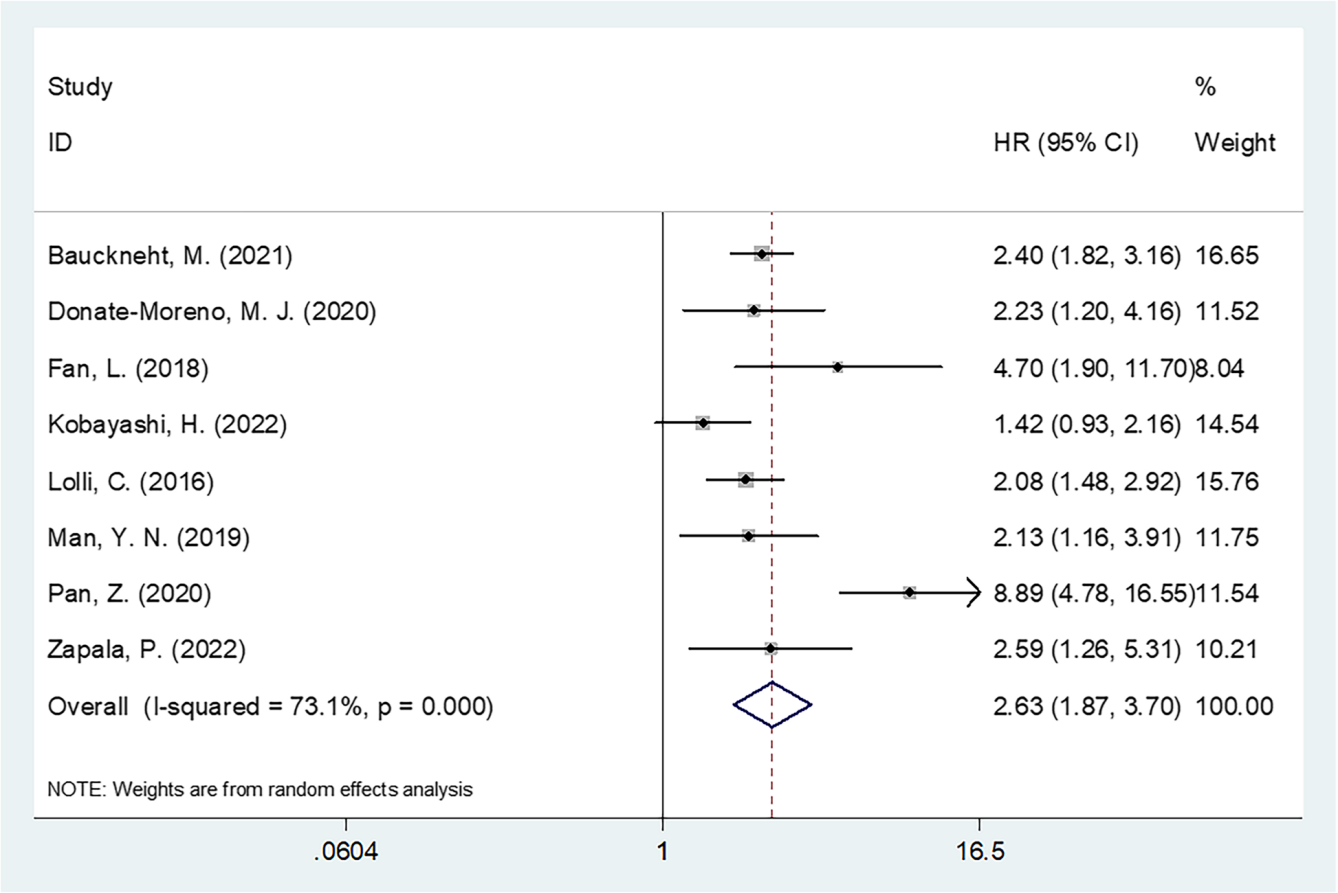
Forest plot of the relationship between high SII and overall survival in prostate cancer

Eight studies with 1803 patients [17–23, 26] provided HR and 95% CI statistics concerning SII for OS. Since the heterogeneity was significant (*I*^2^ = 73.1%, *p* <0.001), we adopted the random-effects model. As is clear from the pooled results in Fig. 2 and Table 2, high SII was linked significantly to the poor OS (*HR* = 2.63, 95% *CI* = 1.87–3.70, *p* < 0.001). According to the subgroup analysis results, a SII elevation remained a prominent prognostic biomarker for OS, irrespective of research design, and investigated center, region, sample size, cutoff, metastatic state, therapeutic management, or type of survival analysis (Table 2).

**Table 2:** Subgroup analysis of the prognostic role of SII for OS in patients with prostate cancer

Subgroup factors	No. of studies	No. of patients	Effects model	HR (95% *CI*)	*p*	Heterogeneity *I*^2^ (%) Ph	
Total	8	1803	Random	2.63 (1.87–3.70)	< 0.001	73.1	< 0.001
Region							
Asian	4	553	Random	3.26 (1.36–7.85)	0.008	88.0	< 0.001
Non-Asian	4	1250	Fixed	2.29 (1.88–2.78)	< 0.001	0	0.910
Sample size							
< 200	5	633	Random	3.00 (1.52–5.93)	0.002	84.1	< 0.001
≥ 200	3	1170	Fixed	2.29 (1.87–2.81)	< 0.001	0	0.767
Metastatic status							
mCRPC	6	1256	Fixed	2.14 (1.80–2.54)	< 0.001	30.8	0.204
Nonmetastatic	2	547	Random	4.87 (1.45–16.29)	0.010	84.5	0.011
Cutoff value							
< 600	4	593	Fixed	2.26 (1.75–2.92)	< 0.001	0	0.430
≥ 600	4	1210	Random	2.89 (1.51–5.53)	0.001	87.0	< 0.001
Cutoff selection							
ROC curve	3	1044	Fixed	2.55 (1.99–3.26)	< 0.001	0	0.382
Literature	2	259	Fixed	2.18 (1.41–3.37)	< 0.001	0	0.919
Median/mean value	2	270	Random	3.50 (0.58–21.13)	0.172	95.6	< 0.001
X-tile software	1	230	-	2.08 (1.48–2.92)	< 0.001	-	-
Study design							
Retrospective	7	1723	Random	2.70 (1.84–3.97)	< 0.001	76.9	< 0.001
Prospective	1	80	-	2.23 (1.20–4.16)	0.012	-	-
Study center							
Single center	7	1284	Random	2.72 (1.75–4.24)	< 0.001	76.9	< 0.001
Multicenter	1	519	-	2.40 (1.82–3.16)	< 0.001	-	-
Treatment							
Hormone therapy + chemotherapy	4	593	Fixed	2.26 (1.75–2.92)	< 0.001	0	0.430
Radical prostatectomy	2	547	Random	4.87 (1.45–16.29)	0.010	84.5	0.011
Radium-223/chemotherapy	2	663	Random	1.89 (1.13–3.16)	0.015	76.0	0.041
Survival analysis type							
Multivariate	6	1493	Random	2.90 (1.78–4.71)	< 0.001	80.1	< 0.001
Univariate	2	310	Fixed	2.11 (1.57–2.85)	< 0.001	0	0.846

*OS* overall survival, *SII* systemic immune-inflammation index, *ROC* receiver operating characteristic, *mCRPC* metastatic castration-resistant prostate cancer

### Prognostic of SII for PFS/bRFS in PCa

**Fig. 3:**
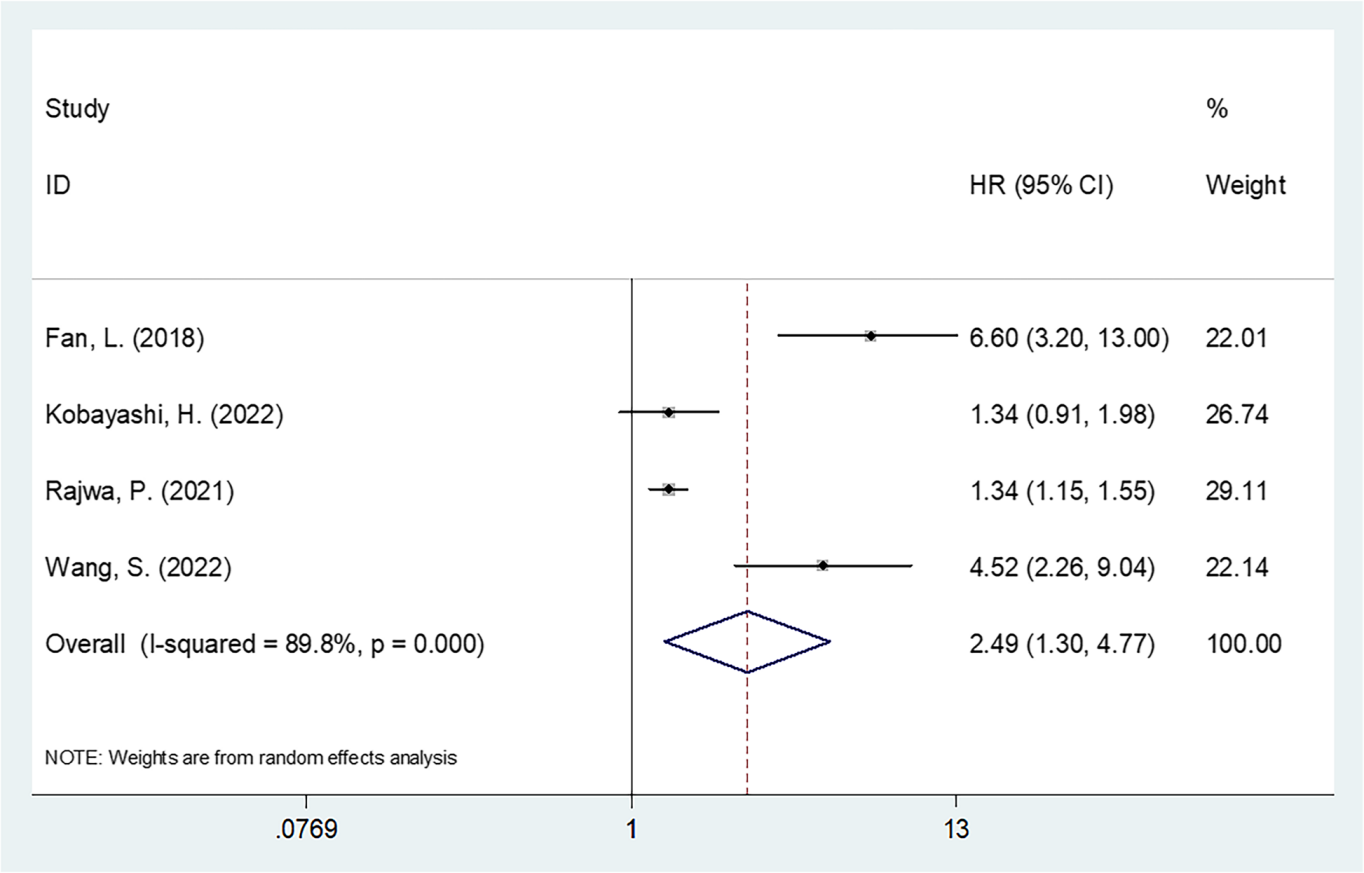
Forest plot of the relationship between high SII and progression-free survival/biochemical recurrence-free survival in prostate cancer

Four studies involving 6578 patients [19, 20, 24, 25] provided data on SII and PFS/bRFS prognosis. According to the pooled HR and 95% CI statistics in Fig. 3 and Table 3, a high SII was a prominent prognostic biomarker for poor PFS/bRFS among the PCa population (*HR* = 2.49, 95% *CI* = 1.30–4.77, *p* = 0.006; *I*^2^ = 89.8%, *Ph* < 0.001). As revealed by the subgroup analysis, region and cutoff were not influencing factors of SII’s prognostic function in PFS/bRFS (Table 3).

**Table 3:** Subgroup analysis of the prognostic role of SII for PFS/bRFS in patients with prostate cancer

Subgroup factors	No. of studies	No. of patients	Effects model	HR (95% *CI*)	*p*	Heterogeneity *I*^2^ (%) Ph	
Total	4	6578	Random	2.49 (1.30–4.77)	0.006	89.8	< 0.001
Region							
Asian	3	539	Random	3.31 (1.14–9.59)	0.027	89.9	< 0.001
Non-Asian	1	6039	-	1.34 (1.15–1.56)	< 0.001	-	--
Sample size							
< 200	2	248	Random	2.89 (0.61–13.79)	0.183	93.4	< 0.001
≥ 200	2	6330	Random	2.34 (0.71–7.69)	0.160	91.2	0.001
Metastatic status							
mCRPC	2	248	Random	2.89 (0.61–13.79)	0.183	93.4	< 0.001
Nonmetastatic	2	6330	Random	2.34 (0.71–7.69)	0.160	91.2	0.001
Cutoff value							
< 600	2	395	Fixed	5.45 (3.33–8.92)	< 0.001	0	0.452
≥ 600	2	6183	Fixed	1.34 (1.17–1.54)	< 0.001	0	1.000
Study center							
Single center	3	539	Random	3.31 (1.14–9.59)	0.027	89.9	< 0.001
Multicenter	1	6039	-	1.34 (1.15–1.56)	< 0.001	-	-
Treatment							
Hormone therapy + chemotherapy	1	104	-	6.60 (3.27–13.30)	< 0.001	-	-
Radical prostatectomy	2	6330	Random	2.34 (0.71–7.69)	0.160	91.2	0.001
Radium-223/chemotherapy	1	144	-	1.34 (0.91–1.98)	0.140	-	-

*PFS* progression-free survival, *SII* systemic immune-inflammation index, *mCRPC* metastatic castration-resistant prostate cancer

### Correlation between SII and clinicopathological factors in PCa

**Fig. 4:**
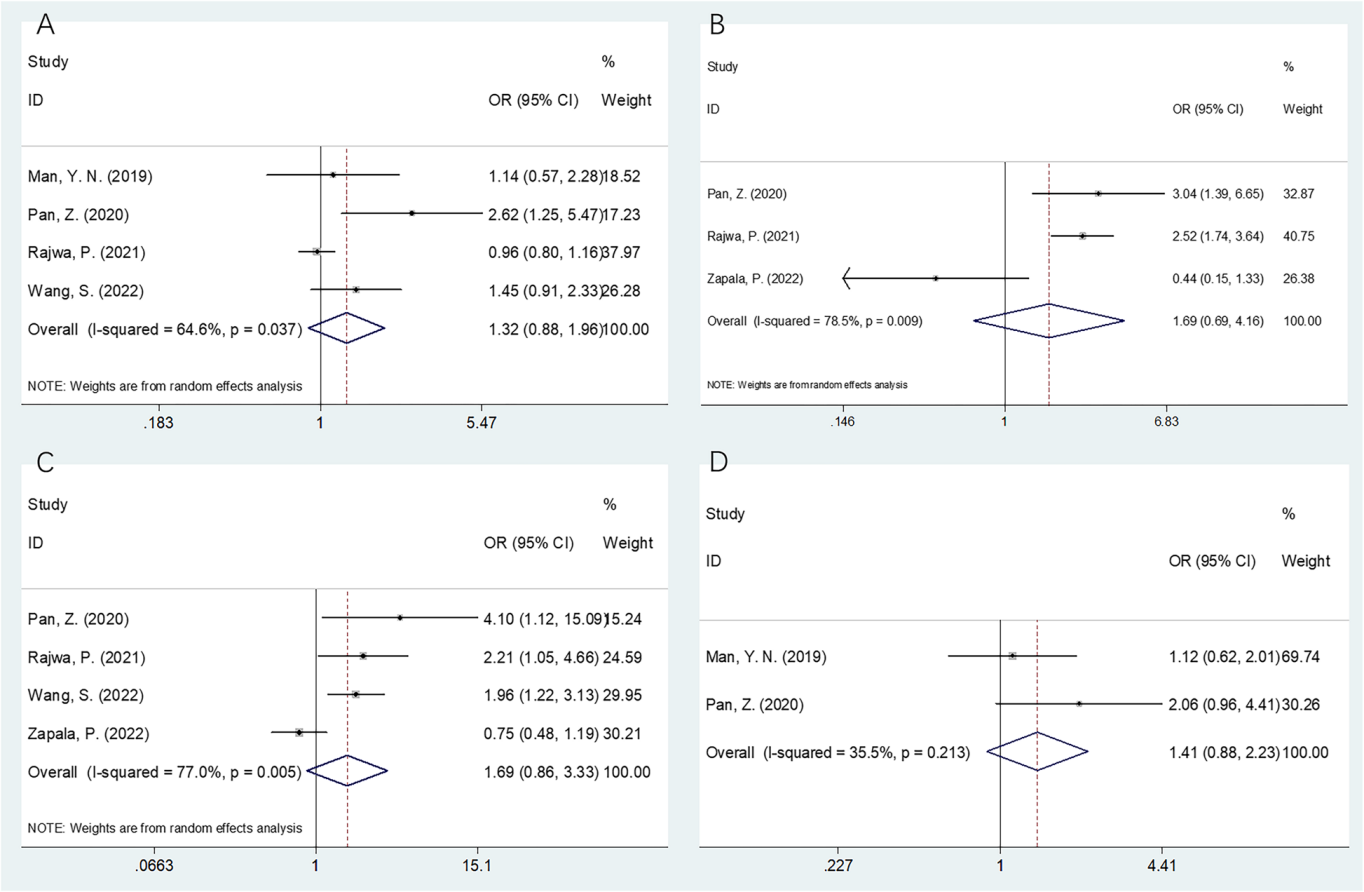
Forest plots of the association between high SII and clinicopathological factors in prostate cancer. **A** Gleason score (≥ 8 vs < 8), **B** lymph node metastasis (yes vs no), **C** T stage (≥ 3 vs 1–2), and **D** age (years) (≥ 70 vs < 70)

Data concerning SII’s association with the clinicopathological parameters in PCa, including the Gleason score (≥ 8 vs. < 8), lymph node (LN) metastasis (yes vs. no), T stage (≥ 3 vs 1–2), and age (≥ 70 vs. < 70) were reported in 5 studies involving 7056 patients [22–26]. As demonstrated by the results in Fig. 4 and Table 4, a high SII was not linked significantly to T stage (*OR* = 1.69, 95% *CI* = 0.86–3.33, *p* = 0.128), the metastasis to LN (*OR* = 1.69, 95% *CI* = 0.69–4.16, *p* = 0.251), age (*OR* = 1.41, 95% *CI* = 0.88–2.23, *p* = 0.150), or the Gleason score (*OR* = 1.32, 95% *CI* = 0.88–1.96, *p* = 0.178).

**Table 4:** The association between SII and clinicopathological features in patients with prostate cancer

Clinicopathological factors	No. of studies	No. of patients	Effects model	OR (95% *CI*)	*p*	Heterogeneity *I*^2^ (%) Ph	
Gleason score (≥ 8 vs < 8)	4	6635	Random	1.32 (0.88–1.96)	0.178	64.6	0.037
Lymph node metastasis (yes vs no)	3	6586	Random	1.69 (0.69–4.16)	0.251	78.5	0.009
T stage (≥ 3 vs 1–2)	4	6877	Random	1.69 (0.86–3.33)	0.128	77.0	0.005
Age (years) (≥ 70 vs < 70)	2	305	Fixed	1.41 (0.88–2.23)	0.150	35.5	0.213

### Sensitivity analysis

**Fig. 5:**
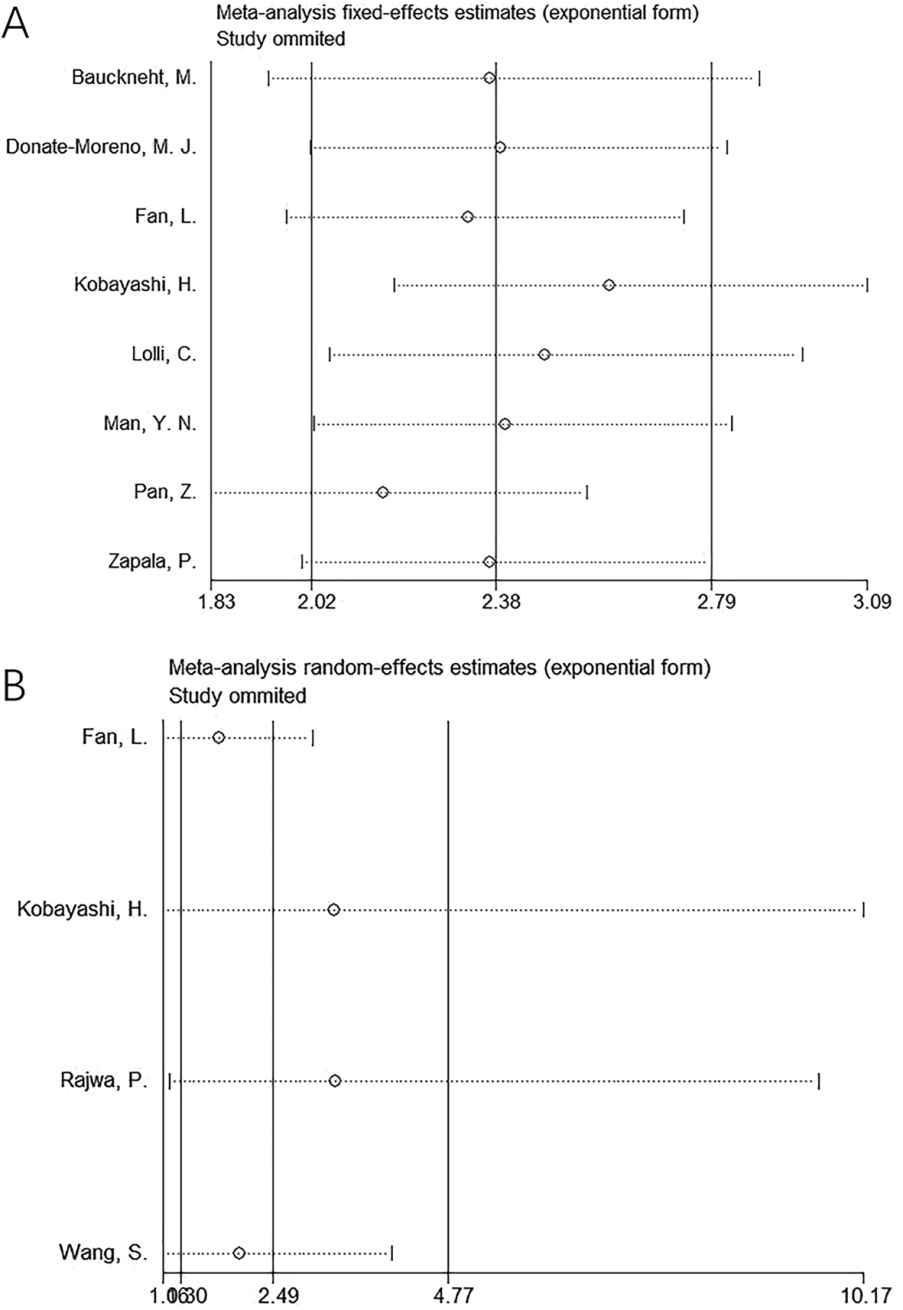
Sensitivity analysis. **A** OS and **B** PFS/bRFS

To test the stability of this meta-analysis, sensitivity analysis was conducted through removing each study in turn to recalculate the combined data. The pooled HR estimates were not significantly changed, indicating that the results were stable (Fig. 5).

### Publication bias

**Fig. 6:**
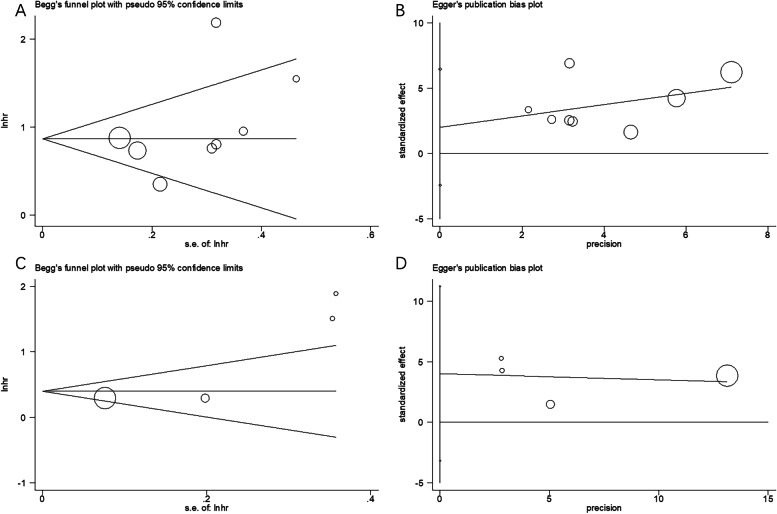
Publication bias determination using Begg’s test and Egger’s test. **A** Begg’s test for OS, *p* = 0.174. **B** Egger’s test for OS, *p* = 0.310. **C** Begg’s test for PFS/bRFS, *p* = 0.089. **D** Egger’s test for PFS/bRFS, *p* = 0.139

Assessment of publication bias was accomplished through the Begg’s and Egger’s tests. However, the publication bias for neither OS (Begg’s test: *p* = 0.174 and Egger’s test: *p* = 0.310) (Fig. 6A and B) nor PFS/bRFS (Begg’s test, *p* = 0.089; Egger’s test, *p* = 0.139) (Fig. 6C and D) was found significant.

## Discussion

In former researches, the potential of SII as a prognostic biomarker has been explored for the PCa population [17–26], despite the unconformable results. In our current meta-analysis, the exact prognostic role of SII in PCa was clarified by pooling the data from 10 studies involving 8133 patients. According to our findings, a high SII represented an independent prognostic biomarker for PFS/bRFS and OS among the PCa patients. Besides, SII exhibited a reliable prognostic power across varying subgroups. However, elevated SII also not correlated with LN metastasis, T stage, Gleason score, or age in PCa. Based on the evidence obtained from this meta-analysis, we recommend the application of SII as a cost-efficient new biomarker for guiding the management and follow-up for the PCa population.

Increasing evidence has suggested the tight linkage of immunoreactions to the cancer occurrence, development, and metastasis [29, 30]. Tumor-derived proinflammatory cytokines like IL (interleukin)-6, IL-8, VEGF, tumor necrosis factor-α, and interferon-γ can be secreted into the tumor microenvironment, leading to chronic inflammation, thus facilitating tumor progression [31]. The SII is a combination of neutrophil, lymphocyte, and platelet counts; thus, it can be elevated in the event of high quantities of neutrophils, platelets, and/or a low quantity of lymphocytes. Current evidence indicates that a microenvironment is offered by the tumor-infiltrating neutrophils, which facilitates the tumor cell growth; they also promote angiogenesis and cell mobility [32]. By constraining the cytolytic potential of various immunocytes, high neutrophil counts inhibit the immunity system [29]. Platelets can release various matrix metalloproteinases to facilitate the degradation of the extracellular matrix, thereby promoting metastasis of cancer cells [33]. In addition, through the proangiogenic cytokine discharge inside the microvasculature of cancer cells, the platelet aggregation is capable of facilitating the tumor growth [34]. In contrast, for the tumor development suppression, lymphocytes are involved critically in the immunosurveillance for cancer [35]. Lymphocytes, including subsets such as CD8+ and CD3+ T cells, correlated with good prognosis in various cancers [36]. Therefore, a high SII is a promising indicator of a combination of neutrophils, lymphocytes, and platelets.

The exact prognostic significance of PSA (prostate-specific antigen), the most extensively applied PCa biomarker [37], in PCa has been reported in several important latest studies [38–40]. A study on 148 patients showed the validity of PSA as a biomarker for forecasting the PCa prognosis when its levels were from 20 to 70 ng/mL [38]. According to another study focusing on PCa sufferers having low levels of PSA, the tumor stage was more advanced when the PSA level at diagnosis was < 3.5 ng/ml compared to that between 3.5 and 10 ng/ml [39]. As suggested by a study enrolling 90 PCa patients whose PSA levels were > 100 ng/mL, the differences in OS or CSS were insignificant among the 3 PSA groups, namely the slightly high (100–200 ng/mL), moderately high (200–1000 ng/mL), and considerably high (> 1000 ng/mL) groups [40]. According to the results of our current meta-analysis, the prognosis of patients was poor when their SII was high. SII is an independent risk factor for PCa and could be a better screening method for PCa before biopsy [41]. Therefore, the SII could be used as a biomarker for improving the diagnostic and prognostic accuracy of PSA. Furthermore, a recent study suggested that the level of CCL2 (serum CC motif (chemokine) ligand 2) is a valid prognostic indicator for poor survival in PCa [42]. Patients with serum CCL2 levels ≥ 320 pg/mL had relatively worse OS, CSS, and CRPC-free survival than those with CCL2 concentrations < 320 pg/mL [42]. The relationship between CCL2 levels and the SII needs to be investigated in future studies.

Notably, this meta-analysis included four studies with the endpoint of PFS [19, 20] and bRFS [24, 25]. Two studies enrolled patients with mCRPC [19, 20], whereas two studies recruited the localized PCa patients [24, 25]. The definition of these endpoints in four studies is not all the same. In Fan’s study [19] with mCRPC patients, PFS referred to an interval from the commencement of the first mCRPC therapy (i.e., docetaxel-prednisone or abiraterone) until the time of radiographic progression. In Kobayashi, H.’s work [20] with mCRPC patients, an interval from the disease diagnosis to progression was regarded as PFS. Besides, disease was considered progressed when the serum level of PSA was elevated by > 2 ng/mL, the rise over nadir was 50%, and/or a new lesion emerged, or the known lesions classified as per the RECIST (ver. 1.1) increased by one or more [43]. In Rajwa’ s study with localized PCa patients, bRFS was defined as the interval from radical prostatectomy to the first PSA rise of two consecutive PSA values > 0.2 ng/ml [24]. Although the definitions are not all the same, our results demonstrated that in PCa, a high SII was a prominent prognostic biomarker for poor PFS/bRFS (Table 3). To probe deeper into SII’s prognostic function among patients with different metastatic status, subgroup analysis was conducted. The results showed that a high SII was not associated with poor PFS/bRFS in localized PCa patients (*p* = 0.160), as well as not with worse PFS/bRFS in mCRPC patients (*p* = 0.183) (Table 3). Because of the relatively small size of samples, our results still need be verified through large-scale researches.

SII’s prominent prognostic value has been pinpointed for varying solid tumors by extensive recent meta-analyses [44–47]. As suggested by a meta-analysis involving 7 studies, an elevated pretreatment SII was linked to inferior carcinoma-specific survival/DFS/PFS and poor OS in pancreatic carcinoma patients [44]. According to another meta-analysis covering 4236 patients, a high pretreatment SII forecasted poor OS in gastric carcinoma [48]. A high SII was also reported to be linked to the poor OS among the renal cell carcinoma sufferers [47]. Through a meta-analysis involving 2,796 patients, Wang et al. reported that elevated pretreatment SII was related to lower OS and earlier time-to-recurrence in hepatocellular carcinoma [49]. As indicated by a latest meta-analysis enrolling 12 studies, high levels of SII were correlated pronouncedly with worse PFS and OS among the colorectal cancer population [50]. Our present results on SII’s prognostic role agree with those in other types of carcinomas.

Regarding several shortcomings of our meta-analysis, first of all, the optimal SII cut-off was not determined. The included studies used different cutoff thresholds, which might have contributed to the heterogeneity among studies. Second, majority of the enrolled studies were retrospective, while there was merely 1 enrolled prospective study. Thus, differences in unadjusted factors could lead to selection bias. Third, our meta-analysis included qualified published studies in English or Chinese only, while failing to enroll relevant articles in other languages, which is also likely to result in inherent heterogeneity.

Conclusively, the present meta-analysis suggests the correlation of an elevated pretreatment SII with the shortened PFS/bRFS and OS among the PCa population. SII monitoring could be a potentially effective approach for improving the survival of patients with PCa.

## Supplementary Information

**Additional file 1.** The PRISMA checklist.

**Additional file 2.** The detailed search strategies for each database.

## Data Availability

The data that support the findings of this study are available from the corresponding author upon reasonable request.

## Abbreviations

SII: Systemic immune-inflammation index
PCa: Prostate cancer
HR: Hazard ratio
CI: Confidence interval
OS: Overall survival
PFS: Progression-free survival
bRFS: Biochemical recurrence-free survival
OR: Odds ratio
mCRPC: Metastatic castration-resistant prostate cancer
CNKI: China National Knowledge Infrastructure
MeSH: Medical Subject Headings
RFS: Recurrence-free survival
DFS: Disease-free survival
NOS: Newcastle-Ottawa scale
Ph: *p*-value of the *Q*-test
IL: Interleukin
PSA: Prostate-specific antigen
CCL2: Chemokine (CC motif) ligand 2
ROC: Receiver operating characteristic
