# Supplementary material for: Prognostic significance of pretreatment systemic immune-inflammation index in patients with prostate cancer: a meta-analysis
Source: World J Surg Oncol. 2023 Jan 5;21:2. doi: 10.1186/s12957-022-02878-7 (PMC9814343; doi:10.1186/s12957-022-02878-7)
Supplement: Supplementary file 2 — Additional file 2. The detailed search strategies for each database. [file 12957_2022_2878_MOESM2_ESM.docx]

**Supplementary file 2. The detailed search strategies for each database.**

**1. Search strategies for PubMed:**

Search: (systemic immune-inflammatory index or SII or systemic-immune-inflammation index or systemic immune-inflammation index) and (prostate cancer or prostate carcinoma or prostatic neoplasms or prostate) Sort by: Most Recent

((("systemic"[All Fields] OR "systemically"[All Fields] OR "systemics"[All Fields]) AND "immune-inflammatory"[All Fields] AND ("abstracting and indexing"[MeSH Terms] OR ("abstracting"[All Fields] AND "indexing"[All Fields]) OR "abstracting and indexing"[All Fields] OR "index"[All Fields] OR "indexed"[All Fields] OR "indexes"[All Fields] OR "indexing"[All Fields] OR "indexation"[All Fields] OR "indexations"[All Fields] OR "indexe"[All Fields] OR "indexer"[All Fields] OR "indexers"[All Fields] OR "indexs"[All Fields])) OR ("stat interface"[Journal] OR "sii"[All Fields]) OR ("systemic-immune-inflammation"[All Fields] AND ("abstracting and indexing"[MeSH Terms] OR ("abstracting"[All Fields] AND "indexing"[All Fields]) OR "abstracting and indexing"[All Fields] OR "index"[All Fields] OR "indexed"[All Fields] OR "indexes"[All Fields] OR "indexing"[All Fields] OR "indexation"[All Fields] OR "indexations"[All Fields] OR "indexe"[All Fields] OR "indexer"[All Fields] OR "indexers"[All Fields] OR "indexs"[All Fields])) OR (("systemic"[All Fields] OR "systemically"[All Fields] OR "systemics"[All Fields]) AND "immune-inflammation"[All Fields] AND ("abstracting and indexing"[MeSH Terms] OR ("abstracting"[All Fields] AND "indexing"[All Fields]) OR "abstracting and indexing"[All Fields] OR "index"[All Fields] OR "indexed"[All Fields] OR "indexes"[All Fields] OR "indexing"[All Fields] OR "indexation"[All Fields] OR "indexations"[All Fields] OR "indexe"[All Fields] OR "indexer"[All Fields] OR "indexers"[All Fields] OR "indexs"[All Fields]))) AND ("prostatic neoplasms"[MeSH Terms] OR ("prostatic"[All Fields] AND "neoplasms"[All Fields]) OR "prostatic neoplasms"[All Fields] OR ("prostate"[All Fields] AND "cancer"[All Fields]) OR "prostate cancer"[All Fields] OR (("prostat"[All Fields] OR "prostate"[MeSH Terms] OR "prostate"[All Fields] OR "prostates"[All Fields] OR "prostatic"[All Fields] OR "prostatism"[MeSH Terms] OR "prostatism"[All Fields] OR "prostatitis"[MeSH Terms] OR "prostatitis"[All Fields]) AND ("carcinoma"[MeSH Terms] OR "carcinoma"[All Fields] OR "carcinomas"[All Fields] OR "carcinoma s"[All Fields])) OR ("prostatic neoplasms"[MeSH Terms] OR ("prostatic"[All Fields] AND "neoplasms"[All Fields]) OR "prostatic neoplasms"[All Fields]) OR ("prostat"[All Fields] OR "prostate"[MeSH Terms] OR "prostate"[All Fields] OR "prostates"[All Fields] OR "prostatic"[All Fields] OR "prostatism"[MeSH Terms] OR "prostatism"[All Fields] OR "prostatitis"[MeSH Terms] OR "prostatitis"[All Fields]))

Translations

systemic: "systemic"[All Fields] OR "systemically"[All Fields] OR "systemics"[All Fields]

index: "abstracting and indexing"[MeSH Terms] OR ("abstracting"[All Fields] AND "indexing"[All Fields]) OR "abstracting and indexing"[All Fields] OR "index"[All Fields] OR "indexed"[All Fields] OR "indexes"[All Fields] OR "indexing"[All Fields] OR "indexation"[All Fields] OR "indexations"[All Fields] OR "indexe"[All Fields] OR "indexer"[All Fields] OR "indexers"[All Fields] OR "indexs"[All Fields]

SII: "Stat Interface"[Journal:__jid101471232] OR "sii"[All Fields]

index: "abstracting and indexing"[MeSH Terms] OR ("abstracting"[All Fields] AND "indexing"[All Fields]) OR "abstracting and indexing"[All Fields] OR "index"[All Fields] OR "indexed"[All Fields] OR "indexes"[All Fields] OR "indexing"[All Fields] OR "indexation"[All Fields] OR "indexations"[All Fields] OR "indexe"[All Fields] OR "indexer"[All Fields] OR "indexers"[All Fields] OR "indexs"[All Fields]

systemic: "systemic"[All Fields] OR "systemically"[All Fields] OR "systemics"[All Fields]

index: "abstracting and indexing"[MeSH Terms] OR ("abstracting"[All Fields] AND "indexing"[All Fields]) OR "abstracting and indexing"[All Fields] OR "index"[All Fields] OR "indexed"[All Fields] OR "indexes"[All Fields] OR "indexing"[All Fields] OR "indexation"[All Fields] OR "indexations"[All Fields] OR "indexe"[All Fields] OR "indexer"[All Fields] OR "indexers"[All Fields] OR "indexs"[All Fields]

prostate cancer: "prostatic neoplasms"[MeSH Terms] OR ("prostatic"[All Fields] AND "neoplasms"[All Fields]) OR "prostatic neoplasms"[All Fields] OR ("prostate"[All Fields] AND "cancer"[All Fields]) OR "prostate cancer"[All Fields]

prostate: "prostat"[All Fields] OR "prostate"[MeSH Terms] OR "prostate"[All Fields] OR "prostates"[All Fields] OR "prostatic"[All Fields] OR "prostatism"[MeSH Terms] OR "prostatism"[All Fields] OR "prostatitis"[MeSH Terms] OR "prostatitis"[All Fields]

carcinoma: "carcinoma"[MeSH Terms] OR "carcinoma"[All Fields] OR "carcinomas"[All Fields] OR "carcinoma's"[All Fields]

prostatic neoplasms: "prostatic neoplasms"[MeSH Terms] OR ("prostatic"[All Fields] AND "neoplasms"[All Fields]) OR "prostatic neoplasms"[All Fields]

prostate: "prostat"[All Fields] OR "prostate"[MeSH Terms] OR "prostate"[All Fields] OR "prostates"[All Fields] OR "prostatic"[All Fields] OR "prostatism"[MeSH Terms] OR "prostatism"[All Fields] OR "prostatitis"[MeSH Terms] OR "prostatitis"[All Fields]

**2. Search strategies for Web of Science:**

ALL=((systemic immune-inflammatory index or SII or systemic-immune-inflammation index or systemic immune-inflammation index) and (prostate cancer or prostate carcinoma or prostatic neoplasms or prostate) )

**3. Search strategies for Embase:**

('systemic immune-inflammatory index' OR (systemic AND 'immune inflammatory' AND ('index'/exp OR index)) OR sii OR 'systemic-immune-inflammation index' OR ('systemic immune inflammation' AND ('index'/exp OR index)) OR 'systemic immune-inflammation index' OR (systemic AND 'immune inflammation' AND ('index'/exp OR index))) AND ('prostate cancer'/exp OR 'prostate cancer' OR (('prostate'/exp OR prostate) AND ('cancer'/exp OR cancer)) OR 'prostate carcinoma'/exp OR 'prostate carcinoma' OR (('prostate'/exp OR prostate) AND ('carcinoma'/exp OR carcinoma)) OR 'prostatic neoplasms'/exp OR 'prostatic neoplasms' OR (prostatic AND ('neoplasms'/exp OR neoplasms)) OR 'prostate'/exp OR prostate)

**4.** **Search strategies for Cochrane Library:**

(systemic immune-inflammatory index or SII or systemic-immune-inflammation index or systemic immune-inflammation index) and (prostate cancer or prostate carcinoma or prostatic neoplasms or prostate) in Title Abstract Keyword

**5. Search strategies for China National Knowledge Infrastructure (CNKI):**

SII and 前列腺

炎症指数 and 前列腺
